# Comparative outcomes of pulmonary artery changes in ductal-patency-dependent pulmonary circulation: Patent ductus arteriosus stent versus modified Blalock–Taussig shunt

**DOI:** 10.1371/journal.pone.0358266

**Published:** 2026-09-15

**Authors:** Panthip Patrakunwiwat, Kraiwat Pattharasripong, Kanjarut Wongwaitaweewong, Supaporn Roymanee, Worakan Promphan, Jirayut Jarutach, Rujira Buntharikpornpun, Pongsanae Duangpakdee, Pimpak Prachasilchai, Suppalak Puttharak

**Affiliations:** 1 Pediatric Heart Center, Queen Sirikit National Institute of Child Health, Department of Medical Services, Ministry of Public Health, Bangkok, Thailand; 2 Division of Pediatric Cardiology, Department of Pediatrics, Faculty of Medicine, Prince of Songkla University, Songkhla, Thailand; 3 Division of Cardiovascular and Thoracic Surgery, Department of Surgery, Faculty of Medicine, Prince of Songkla University, Hat Yai, Songkhla, Thailand; St Paul’s Hospital Millennium Medical College, ETHIOPIA

## Abstract

**Background:**

Neonates with ductal-dependent pulmonary blood flow (DP-PBF) require timely palliation to maintain pulmonary perfusion. Patent ductus arteriosus (PDA) stenting has emerged as a less invasive alternative to modified Blalock–Taussig shunt (mBTS), but comparative data on longitudinal pulmonary artery growth and clinical outcomes remain limited.

**Methods:**

This multicenter retrospective cohort study included infants with DP-PBF who underwent PDA stenting or mBTS as first-stage palliation at Queen Sirikit National Institute of Child Health and Songklanagarind Hospital, Thailand, between January 2017 and December 2022. Pulmonary artery growth was assessed using the McGoon ratio and Nakata index at baseline, 2 months, and 12 months. Longitudinal changes were analyzed using linear mixed-effects models with adjustment for ventricular physiology. Procedural complications were compared, and mortality was evaluated using Kaplan–Meier analysis, Cox regression, and 12-month restricted mean survival time.

**Results:**

Eighty-six infants were included: 41 underwent PDA stenting and 45 underwent mBTS. Baseline pulmonary artery measurements were comparable, although diagnostic composition differed between groups. At 2 months, PDA stenting was associated with greater pulmonary artery growth than mBTS, with a higher McGoon ratio (mean difference, 0.18; 95% CI, 0.05–0.31; p = 0.005) and Nakata index (mean difference, 51.2 mm^2^/m^2^; 95% CI, 23.0–79.5; p < 0.001). By 12 months, the Nakata index was similar between groups (p = 0.90), whereas a statistically significant between-group difference in McGoon ratio remained (mean difference, 0.15; 95% CI, 0.02–0.29; p = 0.028). Procedural complications were less frequent after PDA stenting than after mBTS (17.1% vs 57.8%; risk ratio for mBTS vs PDA stenting, 3.38; 95% CI, 1.65–6.95; p = 0.001). Although mortality was numerically lower after PDA stenting, time-to-event analyses did not show a statistically significant survival difference.

**Conclusions:**

In this cohort, PDA stenting was associated with greater early pulmonary artery growth and fewer observed procedural complications compared with mBTS. By 12 months, the Nakata index was similar between groups, whereas a statistically significant difference in McGoon ratio remained. These findings should be interpreted cautiously because of non-randomized treatment allocation, differences in diagnostic composition, and potential residual confounding.

## Introduction

Congenital heart disease affects approximately 8–10 per 1,000 live births [1,2]. In neonates with ductal patency-dependent pulmonary blood flow (DP-PBF), continuous prostaglandin E1 infusion is commonly used to maintain ductal flow, supporting oxygenation until definitive repair or staged reconstruction [3]. First-stage palliation is therefore undertaken to secure adequate pulmonary perfusion during this interval. Beyond short-term stabilization, a major objective of initial palliation is to promote branch pulmonary artery (PA) growth, as PA dimensions influence the feasibility and complexity of subsequent interventions. The modified Blalock–Taussig systemic-to-pulmonary shunt (mBTS) has traditionally been a standard palliative approach to secure pulmonary blood flow, but it can be associated with operative and postoperative morbidity, including recurrent laryngeal or phrenic nerve injury and thoracic complications such as hemothorax, pneumothorax, and chylothorax [4,5]. Patent ductus arteriosus stenting (PDAs) is a catheter-based alternative to mBTS for initial palliation, offering shorter hospitalization and avoidance of surgery, although stent-specific adverse events, including ductal dissection or rupture, stent thrombosis, stent embolization/migration, and potential branch PA compromise, remain important concerns [6,7].

To date, the comparative literature has not established definitive superiority of PDAs over mBTS with respect to PA growth, a key benchmark for first-stage palliation in neonates with DP-PBF. Although both strategies may promote PA development, the relative contribution of ductal flow-mediated hemodynamics versus surgically created systemic-to-pulmonary shunting remains incompletely defined, and comprehensive head-to-head quantitative data are limited.

Quantitative assessment of PA development is routinely performed during follow-up using established echocardiograms and angiographic indices. The McGoon ratio is defined as the sum of the prebranching diameters of the right and left PAs to the diameter of the descending aorta at the level of the diaphragm, providing an estimate of PA adequacy relative to systemic arterial size [8]. The Nakata index provides a complementary assessment by expressing PA size as the combined cross-sectional area of the right and left PAs normalized to body surface area, with areas derived from measured diameters assuming circular geometry [9]. Together, these complementary metrics provide a structured framework for comparing PA growth following PDAs versus mBTS in neonates with DP-PBF.

The primary objective of this study was to compare pulmonary artery growth between neonates undergoing PDA stenting and those undergoing mBTS for ductal patency-dependent pulmonary blood flow. Secondary objectives were to compare procedural complications and mortality between the two treatment strategies.

## Methods

### Study design and setting

We conducted a multicenter retrospective cohort study including neonates with DP-PBF treated between January 2017 and December 2022 at Queen Sirikit National Institute of Child Health (QSNICH) and Songklanagarind Hospital, Thailand. The study was approved by the institutional ethics committees of both centers (Songklanagarind Hospital: REC.66-499-1-1; Queen Sirikit National Institute of Child Health: REC.070/2567) and conducted in accordance with the Declaration of Helsinki.

Data were accessed for research purposes at Songklanagarind Hospital on 01/01/2024 and at Queen Sirikit National Institute of Child Health (QSNICH) on 01/08/2024, following complete anonymization of all datasets. Throughout the study period, the authors had no access to any information that could identify individual participants.

### Study population

Infants aged <60 days with DP-PBF who underwent first-stage palliation with either a PDA or mBTS were eligible. Patients were excluded from longitudinal growth analyses if they crossed over to an alternative treatment modality after the index procedure or had incomplete PA measurements at the specified follow-up time points. However, all patients undergoing the index procedure were included in analyses of procedural complications and mortality.

### Data collection and follow-up

Baseline demographic and clinical variables were systematically extracted from medical records, including sex, body weight, body length, oxygen saturation, cardiac diagnosis, chromosomal abnormalities, and baseline PA dimensions. PA growth was assessed using the McGoon ratio and Nakata index at baseline, 2 months, and 12 months after the procedure. Branch PA symmetry was evaluated using the left-to-right PA diameter ratio (LPA/RPA) at the same time points.

### Procedural selection and techniques

**Fig 1:**
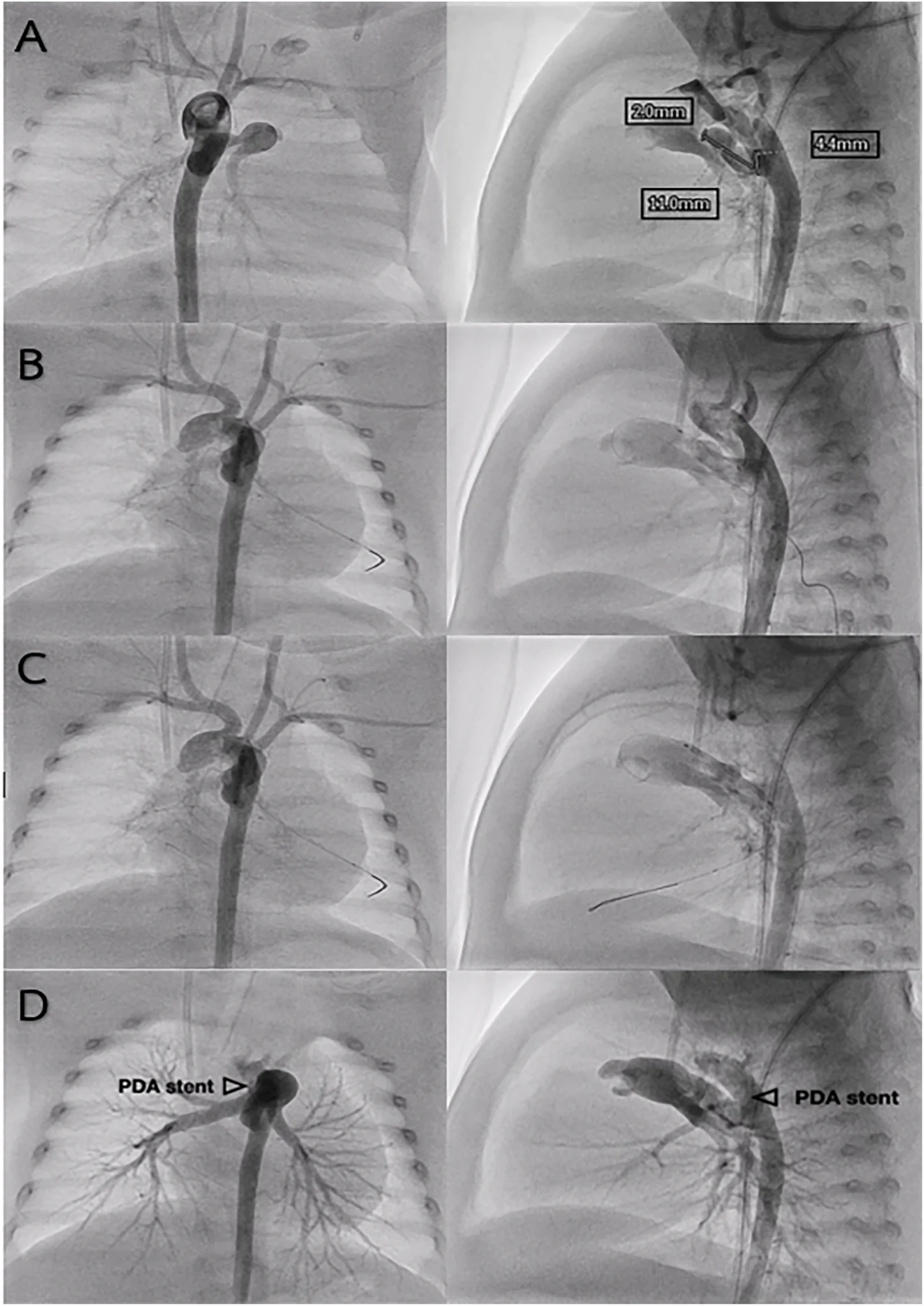
Representative angiographic images from a patient included in the study with pulmonary atresia and intact ventricular septum. (A) Aortography shows a patent ductus arteriosus with a 2-mm pulmonary artery end, 4.4-mm ampulla, and 10-mm length. (B) The ductus is crossed retrogradely with a 0.014-inch guidewire. (C) A Resolute Onyx 4.5 × 15 mm coronary stent is deployed. (D) Completion angiography confirms appropriate stent position without complications.

The choice between PDA stenting and mBTS was determined by the multidisciplinary treating team in accordance with institutional practice, patient anatomy, ventricular physiology, ductal morphology, and procedural feasibility. PDA stenting was generally considered for patients with ductal anatomy suitable for transcatheter intervention, whereas mBTS was preferred in patients with univentricular physiology, markedly tortuous ducts, or any anatomical variation considered unsuitable or high risk for stent implantation. PDA stenting was performed under general anesthesia using standard transcatheter techniques as previously described [6]. Prostaglandin E1 was discontinued for 6–8 hours before the procedure (Fig 1). Ductal anatomy and its relationship to the branch pulmonary arteries were assessed angiographically. A coronary guidewire was advanced across the ductus into a distal pulmonary artery branch, followed by balloon preparation when needed and coronary stent deployment under fluoroscopic guidance. Final angiography confirmed ductal coverage and assessed for branch pulmonary artery compromise.

mBTS was performed using a polytetrafluoroethylene graft anastomosed between the innominate or subclavian artery and the main or branch of the pulmonary artery, with graft size selected based on patient body weight and vessel size. Post-procedural antithrombotic therapy was administered according to institutional practice.

Post-procedural antithrombotic therapy was administered according to institutional practice. Patients undergoing PDA stenting received intravenous heparin for at least 24 hours after the procedure, followed by dual antiplatelet therapy with aspirin and clopidogrel for 6 months. Thereafter, aspirin monotherapy was continued until definitive surgical repair or the next-stage palliation. In contrast, patients undergoing mBTS received intravenous heparin for at least 24 hours postoperatively, followed by aspirin monotherapy at 3–5 mg/kg/day until definitive surgical repair or the next-stage palliation.

### Outcomes

The primary outcome was PA growth, assessed by changes in McGoon ratio and Nakata index at 2 and 12 months. Secondary outcomes included procedural complications and all-cause mortality.

### Sample size estimation

Sample size was estimated for the primary outcome of pulmonary artery growth, assessed by the Nakata index. Using a two-independent-means formula with a two-sided test, prior reference data reported mean Nakata indices of 162 ± 26 and 194.7 ± 48 in infants undergoing ductal stenting and surgical shunting, respectively [10]. Assuming a two-sided alpha level of 0.05, 80% power, and a 2:1 allocation ratio, the required sample size was 41 patients. After accounting for approximately 10% incomplete data, the final estimated sample size was 46 patients.

### Statistical analysis

Categorical variables are presented as frequencies and percentages and were compared using the χ² test or Fisher’s exact test, as appropriate. Continuous variables were assessed for approximate normality and are presented as mean ± standard deviation (SD). Between-group comparisons of continuous variables were performed using two-sided independent-samples t tests. Diagnosis distributions were compared using the χ² test. Differences in PA indices across diagnostic categories were evaluated using two-sided one-way analysis of variance (ANOVA) at baseline and 12 months, with Bonferroni-adjusted post hoc comparisons. For longitudinal assessment of PA growth, the McGoon ratio and Nakata index were compared between the PDAs and mBTS groups at each prespecified time point (baseline, 2 months, and 12 months) using linear mixed-effects (random-intercept) models, with treatment group, follow-up time, and their interaction as fixed effects and a random intercept for each patient. This repeated-measures approach used all available observations under a missing-at-random assumption, thereby accommodating incomplete follow-up without restricting the complete cases. Between-group differences (PDAs minus mBTS) at each time point were estimated as model-based linear contrasts and reported with 95% confidence intervals; the overall group-by-time interaction was tested with a likelihood-ratio test. To address potential confounding by indication, the longitudinal models were repeated with additional adjustment for ventricular physiology (biventricular vs univentricular) and its interaction with time. Analyses of branch pulmonary artery symmetry (LPA/RPA ratio) were performed among patients with available measurements at each time point.

Procedural complications and mortality were compared using the χ² test. Risk ratios (RRs) with 95% confidence intervals (CIs) were calculated to quantify the relative risk associated with mBTS compared with PDAs.

Mortality was evaluated descriptively and using time-to-event analyses. Overall survival was defined as the time from the index procedure to death from any cause or censoring at the last documented follow-up. Because the index procedure date, date of death, and censoring or last follow-up date were available, Kaplan–Meier curves were generated and compared using the log-rank test. Cox proportional hazards models were used to estimate hazard ratios for mortality, with additional adjustment for ventricular physiology. The proportional hazards assumption was assessed before Cox regression. Restricted mean survival time was calculated up to 12 months to summarize survival over the prespecified follow-up interval.

To further evaluate potential confounding due to differential diagnostic composition and loss to follow-up, sensitivity analyses were performed restricting the cohort to patients with complete longitudinal data and stratifying by ventricular physiology. The direction and magnitude of between-group differences were compared with the primary analysis to assess consistency. All analyses were conducted using Stata version 19.5 (StataCorp LLC, College Station, TX, USA). A two-sided *p-value* < 0.05 was considered statistically significant.

## Results

**Fig 2:**
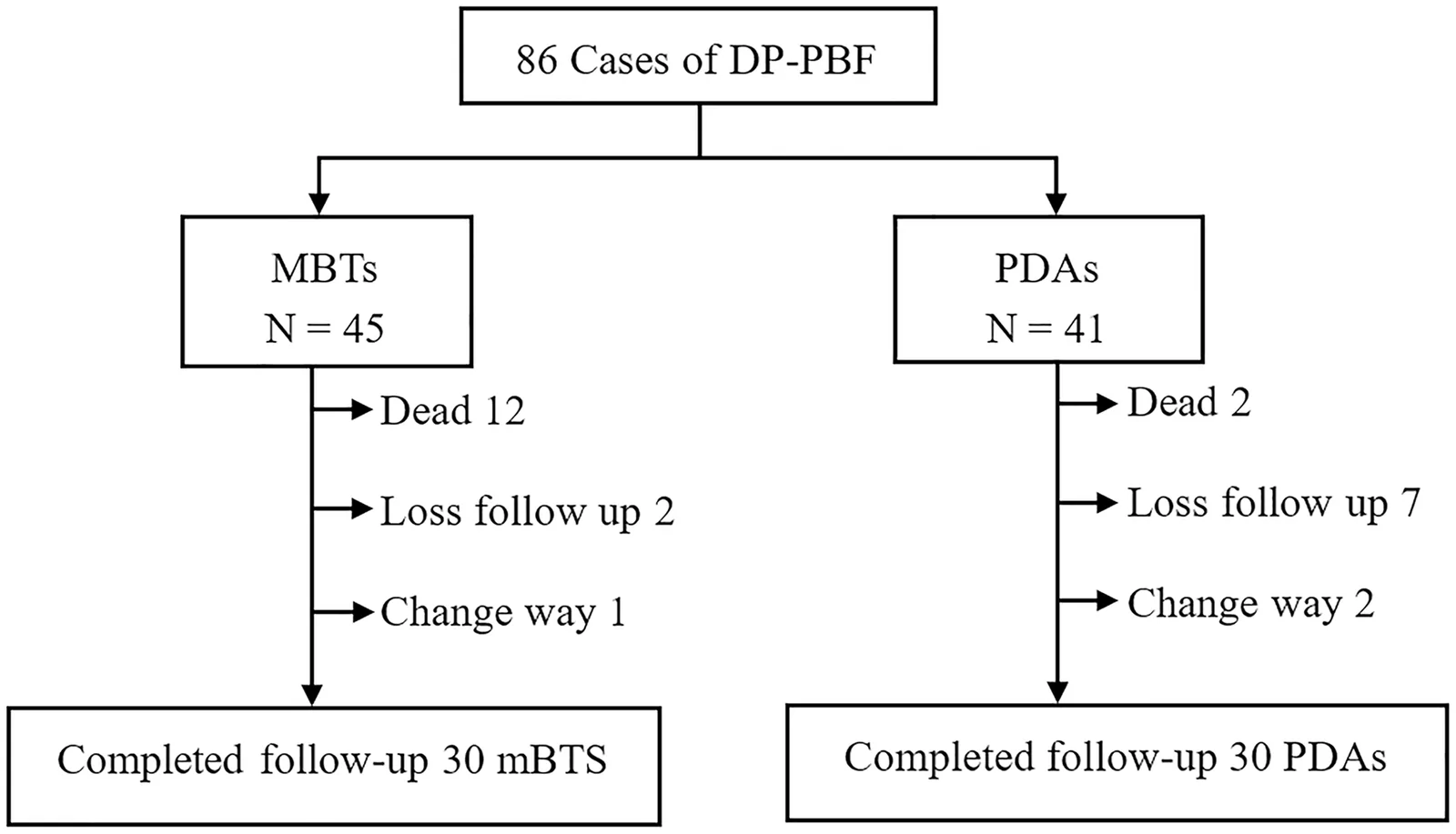
Initial surgical and interventional strategies for duct patency-dependent pulmonary blood flow.

A total of 86 infants with DP-PBF were included; 45 received an mBTS, and 41 underwent PDAs as first-stage palliation. During follow-up, attrition in the mBTS group was due to mortality (n = 12), loss to follow-up (n = 2), and conversion to an alternative treatment modality (n = 1), leaving 30 patients with complete follow-up. In the PDAs group, attrition was due to mortality (n = 2), loss to follow-up (n = 7), and conversion to an alternative treatment modality (n = 2), resulting in 30 patients with complete follow-up for the final analysis (Fig 2).

Table 1 presents baseline characteristics of the 86 infants with DP-PBF included in the analysis. The proportion of male infants was similar between groups. Mean weight was comparable, whereas mean body height was greater in the PDAs group *(p =* 0.002). Baseline oxygen saturation did not differ significantly between groups. Chromosomal abnormalities were infrequent and comparable between the two groups. The distribution of underlying diagnoses differed between groups (*p* < 0.001), with univentricular heart disease more frequent in the MBTs group and pulmonary atresia with an intact ventricular septum more frequent in the PDAs group; pulmonary atresia with a ventricular septal defect and Tetralogy of Fallot were less common in both groups. Baseline PA measurements were similar, including LPA diameter, RPA diameter, McGoon ratio, and Nakata index.

**Table 1:** Comparative characteristics of patients in the PDAs and mBTS groups.

	Total N = 86	PDAs N = 41	mBTS N = 45	P-value	95% CI
**Male**	56 (65.12%)	25 (60.98%)	31 (68.89%)	0.442	
**Weight** kg, mean (SD)	3.217 (0.463)	3.262 (0.486)	3.176 (0.442)	0.392	3.118–3.317
**Length** cm, mean (SD)	49.808 (2.328)	50.610 (2.201)	49.078 (2.218)	0.002	49.309–50.307
**Oxygen**_**s**_ %, mean (SD)	82.488 (7.041)	81.244 (8.428)	83.622 (5.331)	0.118	80.979–83.998
**Chromosome abnormality**	4 (4.65%)	1 (2.44%)	3 (6.67%)	0.352	
**Diagnostics**					
UVH	39 (45.35%)	13 (31.71%)	26 (57.78%)	<0.001	
PA/IVS	22 (25.58%)	20 (48.78%)	2 (4.44%)		
PA/VSD	16 (18.60%)	6 (14.63%)	10 (22.22%)		
TOF	9 (10.84%)	2 (4.88%)	7 (15.56%)		
**LPA mm,** mean (SD)	4.153 (0.916)	4.231(1.085)	4.080 (0.735)	0.453	3.957–4.350
**RPA mm,** mean (SD)	4.135 (0.930)	4.262 (1.053)	4.02 (0.795)	0.230	3.936–4.335
**McGoon ratio** mean (SD)	1.440 (0.265)	1.433 (0.311)	1.446 (0.218)	0.827	1.383–1.497
**Nakata index** mm²/m², mean (SD)	134.321 (52.923)	140.075(62.432)	129.206 (42.843)	0.348	122.905–145.736

Oxygen_s_; oxygen saturation, PA/IVS; pulmonary atresia with intact ventricular septum, TOF; Tetralogy of Fallot, PA/VSD; pulmonary atresia with ventricular septal defect, UVH; univentricular heart disease, TGA/VSD/VPS; transposition of great artery with ventricular septal defect and valvular pulmonary stenosis, RPA; right pulmonary artery, LPA; left pulmonary artery.

Baseline diagnostic composition differed between procedural groups (*p* < 0.001). The McGoon ratio did not differ across diagnostic categories at baseline (one-way ANOVA: F (3,82) = 0.47, *p* = 0.7038) or at 12 months (F (3,63) = 2.31, *p* = 0.0847); Bonferroni-adjusted pairwise comparisons were not statistically significant (baseline: all adjusted *p* = 1.000; 12 months: all adjusted *p* ≥ 0.073). Similarly, the Nakata index did not differ across diagnostic categories at baseline (F (3,81) = 1.15, *p* = 0.3345) or at 12 months (F (3,63) = 0.57, *p* = 0.6374), with no significant Bonferroni-adjusted post hoc differences (baseline: all adjusted *p* ≥ 0.540; 12 months: all adjusted *p* = 1.000).

**Fig 3:**
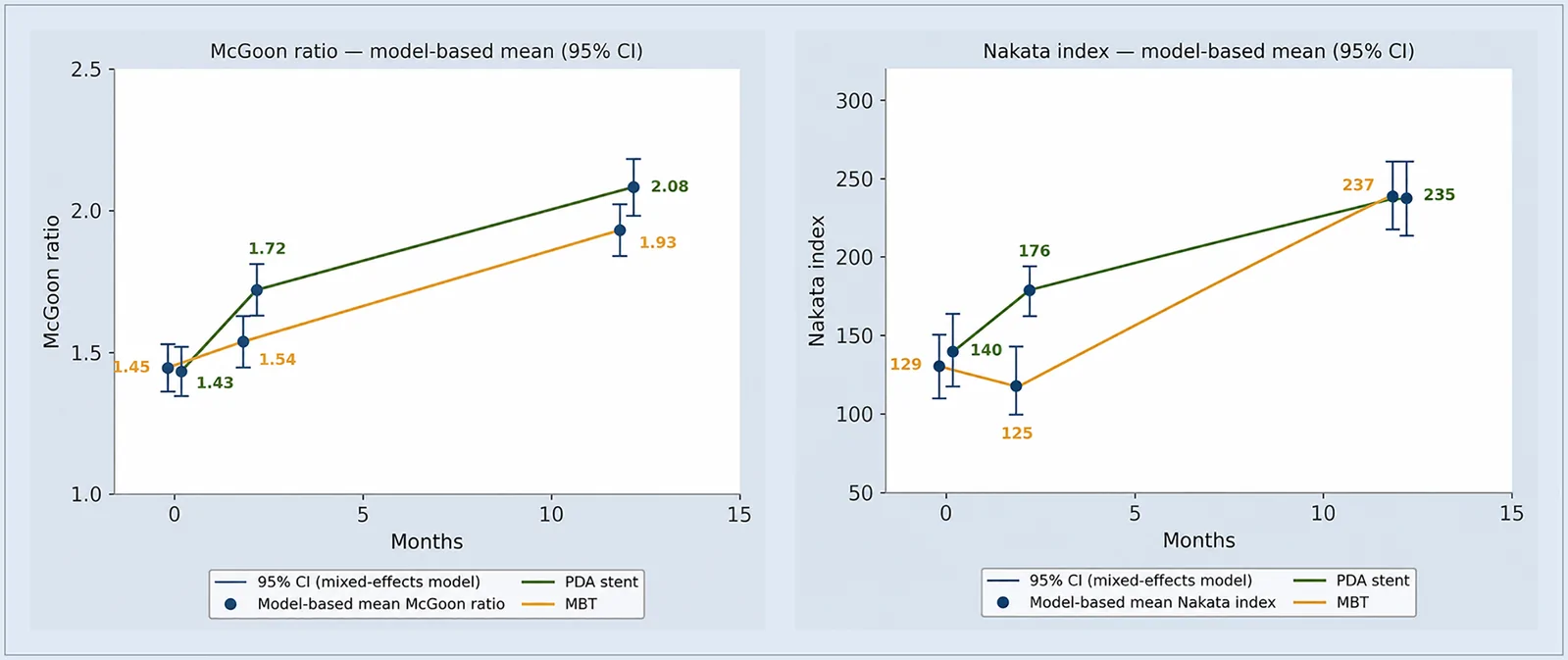
Longitudinal changes in pulmonary artery growth after PDA stenting and mBTS. (A) McGoon ratio and (B) Nakata index were assessed at baseline, 2 months, and 12 months after initial palliation. Points represent model-based mean estimates derived from linear mixed-effects models, and error bars indicate 95% confidence intervals.

Pulmonary artery growth was evaluated using the McGoon ratio and Nakata index at baseline, 2 months, and 12 months after PDA stenting or mBTS (Fig 3 and Table 2). The mean McGoon ratio was 1.450 ± 0.285 at baseline. At baseline, the model-based between-group difference, calculated as PDA stenting minus mBTS, was −0.01 (95% CI, −0.13 to 0.11; p = 0.84). At 2 months, the mean McGoon ratio was 1.721 ± 0.325 in the PDA stenting group and 1.540 ± 0.194 in the mBTS group, with a model-based between-group difference of 0.18 (95% CI, 0.05 to 0.31; p = 0.005). At 12 months, the mean McGoon ratio was 2.080 ± 0.323 in the PDA stenting group and 1.930 ± 0.334 in the mBTS group, with a model-based between-group difference of 0.15 (95% CI, 0.02 to 0.29; p = 0.028). The longitudinal change in the McGoon ratio differed between treatment groups (likelihood-ratio p = 0.018). After adjustment for ventricular physiology, the corresponding between-group differences were 0.00 at baseline (95% CI, −0.12 to 0.13; p = 0.97), 0.19 at 2 months (95% CI, 0.06 to 0.32; p = 0.005), and 0.16 at 12 months (95% CI, 0.02 to 0.30; p = 0.023).

**Table 2:** Between-group differences in pulmonary artery growth (PDAs minus mBTS) from linear mixed-effects models, with 95% confidence intervals.

Outcome/ time point	Unadjusted difference (95% CI)	*p*	Adjusted† difference (95% CI)	*p*
**McGoon ratio**				
Baseline	−0.01 (−0.13 to 0.11)	0.84	0.00 (−0.12 to 0.13)	0.97
2 months	0.18 (0.05 to 0.31)	0.005	0.19 (0.06 to 0.32)	0.005
12 months	0.15 (0.02 to 0.29)	0.028	0.16 (0.02 to 0.30)	0.023
**Nakata index (mm²/m²)**				
Baseline	10.9 (−15.7 to 37.4)	0.42	8.0 (−19.5 to 35.6)	0.57
2 months	51.2 (23.0 to 79.5)	<0.001	51.9 (22.8 to 81.0)	<0.001
12 months	−2.0 (−31.7 to 27.8)	0.90	−3.5 (−34.4 to 27.5)	0.83

Differences are PDAs minus mBTS estimated as model-based contrasts from linear mixed-effects models with a random intercept for each patient. †Adjusted models additionally include ventricular physiology (biventricular vs univentricular) and its interaction with time. Overall group-by-time interaction (likelihood-ratio test): McGoon ratio p = 0.018; Nakata index p = 0.009. CI, confidence interval; PDAs, patent ductus arteriosus stent; mBTS, modified Blalock–Taussig shunt.

The mean baseline Nakata index in the overall cohort was 134.322 ± 52.923 mm^2^/m^2^ (Table 1). At baseline, the model-based between-group difference was 10.9 mm^2^/m^2^ (95% CI, −15.7 to 37.4; p = 0.42). At 2 months, the mean Nakata index was 176.612 ± 58.080 mm^2^/m^2^ in the PDA stenting group and 124.287 ± 41.292 mm^2^/m^2^ in the mBTS group, with a model-based between-group difference of 51.2 mm^2^/m^2^ (95% CI, 23.0 to 79.5; p < 0.001). At 12 months, the mean Nakata index was 237.537 ± 79.135 mm^2^/m^2^ in the PDA stenting group and 236.569 ± 84.913 mm^2^/m^2^ in the mBTS group, with a model-based between-group difference of −2.0 mm^2^/m^2^ (95% CI, −31.7 to 27.8; p = 0.90). The longitudinal change in the Nakata index differed between treatment groups (likelihood-ratio p = 0.009). After adjustment for ventricular physiology, the corresponding between-group differences were 8.0 mm^2^/m^2^ at baseline (95% CI, −19.5 to 35.6; p = 0.57), 51.9 mm^2^/m^2^ at 2 months (95% CI, 22.8 to 81.0; p < 0.001), and −3.5 mm^2^/m^2^ at 12 months (95% CI, −34.4 to 27.5; p = 0.83). The LPA/RPA ratio did not differ at baseline or at 2 months but was significantly lower in the PDAs group at 12 months (Table 3), indicating progressive branch asymmetry despite similar global PA dimensions.

**Table 3:** Comparison of branch pulmonary artery symmetry (LPA/RPA ratio) between PDAs and mBTS groups.

Time point	PDAs	mBTS	Mean difference	95% CI	*p-value*
	mean ± SD	mean ± SD	(PDAs − mBTS)		
Baseline	0.999 ± 0.130	1.035 ± 0.170	−0.036	−0.073–0.001	0.058
2 months	0.968 ± 0.187	1.075 ± 0.711	−0.107	−0.242–0.029	0.122
12 months	0.919 ± 0.167	0.994 ± 0.217	−0.075	−0.130–0.020	0.008

*p-values were calculated using a two-sample t test (two-sided) comparing PDAs vs mBTS at each time point. SD, standard deviation; PDAs, patent ductus arteriosus stent; mBTS, modified Blalock–Taussig shunt.

**Fig 4:**
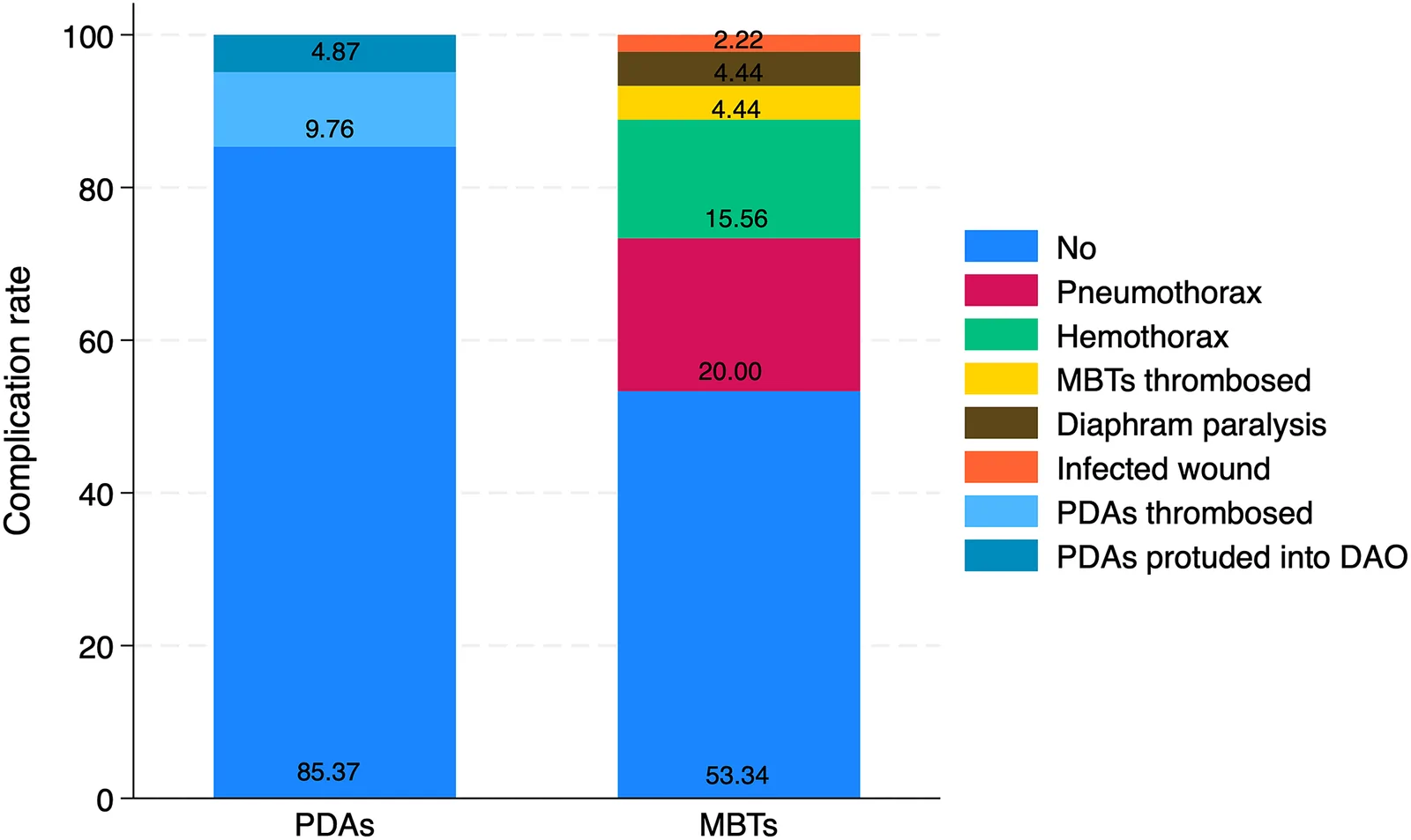
Comparative distribution of procedural complications following PDAs and mBTS.

Overall, complications were observed in 33 of 86 patients (38.4%). Complications occurred in 7 of 41 patients (17.1%) in the PDA stenting group and 26 of 45 patients (57.8%) in the mBTS group. The unadjusted risk ratio for complications, calculated as mBTS versus PDA stenting, was 3.38 (95% CI, 1.65–6.95; p = 0.001). The distribution of complication subtypes is shown in Fig 4. In the PDA stenting group, PDA thrombosis occurred in 4 patients (9.76%) and stent protrusion into the descending aorta occurred in 2 patients (4.87%). In the mBTS group, pneumothorax occurred in 9 patients (20.0%), hemothorax in 7 patients (15.6%), shunt thrombosis in 2 patients (4.44%), diaphragmatic paralysis in 2 patients (4.44%), and wound infection in 1 patient (2.22%) (Fig 4).

**Fig 5:**
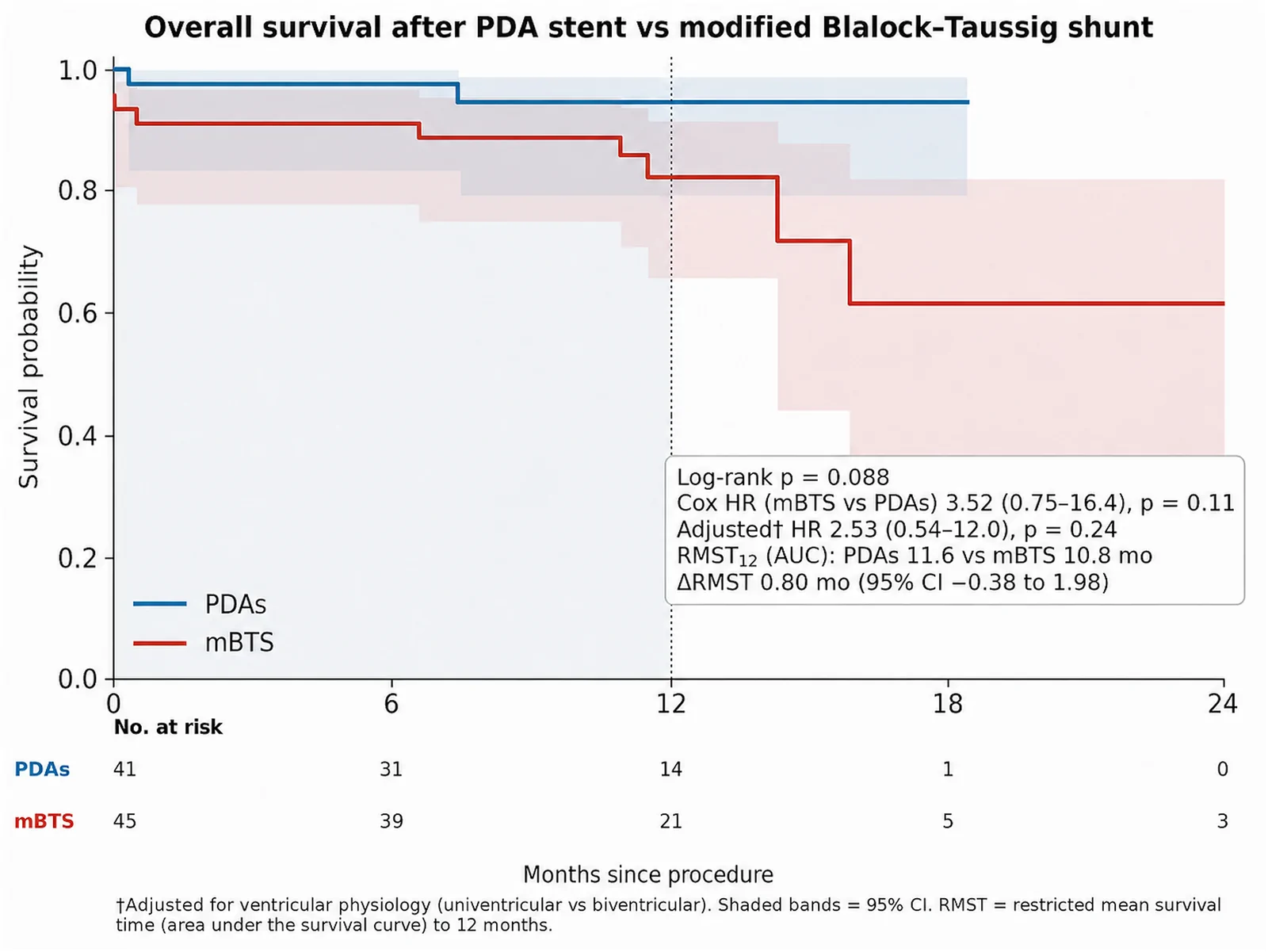
Kaplan–Meier analysis of overall survival after PDA stenting and mBTS.

All-cause death occurred in 14 of 86 patients (16.3%): 2 of 41 patients (4.9%) in the PDA stenting group and 12 of 45 patients (26.7%) in the mBTS group. The log-rank test comparing Kaplan–Meier survival curves showed p = 0.088. In the unadjusted Cox proportional hazards model, the hazard ratio for mortality in the mBTS group compared with the PDA stenting group was 3.52 (95% CI, 0.75–16.4; p = 0.11). After adjustment for ventricular physiology, the hazard ratio was 2.53 (95% CI, 0.54–12.0; p = 0.24). The 12-month restricted mean survival time was 11.6 months in the PDA stenting group and 10.8 months in the mBTS group, with a between-group difference of 0.80 months (95% CI, −0.38 to 1.98) (Fig 5).

## Discussion

Neonates with DP-PBF require prompt intervention to maintain pulmonary blood flow because systemic oxygenation depends on ductal patency. Since the early 1990s, PDAs have been used as a feasible alternative to surgical shunts and may provide balanced perfusion to both PAs in the short term [11]. mBTS remains a widely used palliative procedure, particularly for patients expected to follow a single-ventricle pathway [12]. In our retrospective cohort, PA growth assessed by the McGoon ratio and Nakata index did not differ between PDAs and mBTS, consistent with prior comparative studies reporting similar PA growth after these strategies [13,14]^.^

### Pulmonary artery growth and branch symmetry

In this repeated-measures analysis, PDA stenting was associated with greater early pulmonary artery growth than mBTS, as reflected by significantly higher McGoon ratios and Nakata indices at 2 months. This early difference may be clinically relevant because the 2-month interval corresponds to early reassessment after initial palliation, when adequacy of pulmonary blood flow, need for closer surveillance or reintervention, and planning for subsequent surgical repair or next-stage palliation are evaluated.

By 12 months, the Nakata index was similar between groups, whereas a statistically significant between-group difference in McGoon ratio remained. These findings suggest that PDA stenting was associated with greater early pulmonary artery growth, followed by attenuation of the between-group difference over time. The similar Nakata index at 12 months indicates largely comparable indexed pulmonary artery size, while the remaining McGoon ratio difference may reflect residual differences in branch pulmonary artery dimensions relative to the descending aorta. Therefore, the 12 month findings should be interpreted as partial convergence of pulmonary artery growth rather than complete equivalence between treatment groups.

In our practice, post-procedural antithrombotic regimens differed between groups, with dual antiplatelet therapy used after PDA stenting and aspirin monotherapy used after mBTS. Late lumen reduction related to neointimal proliferation may reduce effective pulmonary blood flow after ductal stenting and could attenuate early gains in pulmonary artery growth over time. Supporting this hypothesis, Al Kindi et al. used four-dimensional flow magnetic resonance imaging after PDA stenting and demonstrated reduced indexed branch pulmonary artery flow at follow-up, consistent with evolving stent-related flow limitation. However, our study was not designed to evaluate this mechanism directly; therefore, this explanation should be considered hypothesis-generating [15]. Adjustment for ventricular physiology did not materially change the estimates, suggesting that ventricular physiology alone did not account for the observed differences. Nevertheless, because treatment allocation was non-randomized and may have been influenced by ductal morphology, anatomic complexity, and procedural feasibility, residual confounding remains possible. The results should therefore be interpreted as associative, and not as evidence of causal superiority of PDA stenting over mBTS.

Assessment of branch pulmonary artery symmetry provided additional information beyond the global pulmonary artery indices. In our cohort, the LPA/RPA ratio did not differ significantly between groups during early follow-up but was significantly lower in the PDA stenting group at 12 months. This suggests that branch pulmonary artery remodeling may evolve, even when indexed pulmonary artery size, as measured by the Nakata index, becomes similar between groups. Prior studies have reported variable findings. Santoro et al. found comparable overall pulmonary artery growth after ductal stenting and surgical shunting, while emphasizing the influence of ductal anatomy, ductal insertion, and pulmonary blood flow distribution on branch pulmonary artery balance [13]. In contrast, Glatz et al. reported greater growth in pulmonary artery branches, and improved symmetry after ductal stenting [16]. Our findings suggest that global indices may not fully capture branch-specific remodeling and support longitudinal surveillance of both conduit patency and branch pulmonary artery symmetry after palliation.

### Procedural outcomes and clinical implications

In this cohort, procedural complications were observed more frequently after mBTS than after PDA stenting, and the pattern of complications differed between treatment strategies. Complications after PDA stenting were primarily stent-related, whereas complications after mBTS were more commonly surgery-related, including pleural and thoracic complications. Although crude mortality was also higher in the mBTS group, this finding should be interpreted with caution as time-to-event analyses did not demonstrate a statistically significant survival difference. Kaplan–Meier analysis, Cox proportional hazards modeling, and 12-month restricted mean survival time analysis showed a higher observed survival probability after PDA stenting, but the between-group differences were not statistically significant.

These findings are consistent with prior comparative studies by McMullan et al. [17], Amoozgar, et al. [14], and Valencia et al. [18], which generally reported broadly comparable early survival after PDA stenting and surgical shunting, without clear evidence of mortality superiority for either strategy. In our institutional practice, mBTS was more commonly selected for patients with univentricular physiology, markedly tortuous ductal morphology, or any anatomical variations considered unsuitable or high-risk for PDA stenting. Therefore, the observed differences in complications and crude mortality may partly reflect baseline anatomical complexity, physiological instability, procedural feasibility, and preprocedural risk rather than a direct treatment effect. Accordingly, the survival findings should be interpreted as observational associations and not as evidence of a causal survival advantage of PDA stenting over mBTS. Future prospective studies with standardized treatment selection, adequate adjustment for ductal morphology and ventricular physiology, and uniform follow-up are needed to better define the independent effect of palliation strategy on procedural safety and survival.

### Limitations

Because this was a retrospective study, the timing of some non-fatal events, including complications and reinterventions, was not consistently available. Time-to-event analyses were therefore performed for mortality, whereas other clinical outcomes were summarized over the follow-up period.

## Conclusion

PDA stenting was associated with greater early pulmonary artery growth and fewer procedural complications in this cohort; however, interpretation is limited by non-randomized treatment allocation and baseline differences in anatomical complexity.

## References

[1] van der Linde D, Konings EEM, Slager MA, Witsenburg M, Helbing WA, Takkenberg JJM, et al. Birth prevalence of congenital heart disease worldwide: a systematic review and meta-analysis. J Am Coll Cardiol. 2011;58(21):2241–7. doi: 10.1016/j.jacc.2011.08.025 22078432

[2] Hoffman JIE, Kaplan S. The incidence of congenital heart disease. J Am Coll Cardiol. 2002;39(12):1890–900. doi: 10.1016/s0735-1097(02)01886-7 12084585

[3] Akkinapally S, Hundalani SG, Kulkarni M, Fernandes CJ, Cabrera AG, Shivanna B, et al. Prostaglandin E1 for maintaining ductal patency in neonates with ductal-dependent cardiac lesions. Cochrane Database Syst Rev. 2018;2(2):CD011417. doi: 10.1002/14651858.CD011417.pub2 29486048 PMC6491149

[4] Sasikumar N, Hermuzi A, Fan C-PS, Lee K-J, Chaturvedi R, Hickey E, et al. Outcomes of Blalock-Taussig shunts in current era: a single center experience. Congenit Heart Dis. 2017;12(6):808–14. doi: 10.1111/chd.12516 28736841

[5] Glatz AC, Petit CJ, Goldstein BH, Kelleman MS, McCracken CE, McDonnell A, et al. Comparison between patent ductus arteriosus stent and modified Blalock-Taussig shunt as palliation for infants with ductal-dependent pulmonary blood flow: insights from the congenital catheterization research collaborative. Circulation. 2018;137(6):589–601. doi: 10.1161/CIRCULATIONAHA.117.029987 29042354

[6] Promphan W, Qureshi SA. Technical modifications for ductal stenting in neonates with duct-dependent pulmonary circulation. Hearts. 2021;2(2):188–201. doi: 10.3390/hearts2020015

[7] Helal AM, Elmahrouk AF, Bekheet S, Barnawi HI, Jamjoom AA, Galal MO, et al. Patent ductus arteriosus stenting versus modified Blalock-Taussig shunt for palliation of duct-dependent pulmonary blood flow lesions. J Card Surg. 2022;37(9):2571–80. doi: 10.1111/jocs.16692 35726659

[8] Fontan F, Fernandez G, Costa F, Naftel DC, Tritto F, Blackstone EH, et al. The size of the pulmonary arteries and the results of the Fontan operation. J Thorac Cardiovasc Surg. 1989;98(5 Pt 1):711–9; discussion 719-24. doi: 10.1016/s0022-5223(19)34293-x 2811408

[9] Nakata S, Imai Y, Takanashi Y, Kurosawa H, Tezuka K, Nakazawa M, et al. A new method for the quantitative standardization of cross-sectional areas of the pulmonary arteries in congenital heart diseases with decreased pulmonary blood flow. J Thorac Cardiovasc Surg. 1984;88(4):610–9. doi: 10.1016/s0022-5223(19)38300-x 6482493

[10] Nasser BA, Abdulrahman M, Qwaee AAL, Alakfash A, Mohamad T, Kabbani MS. Impact of stent of ductus arteriosus and modified Blalock-Taussig shunt on pulmonary arteries growth and second-stage surgery in infants with ductus-dependent pulmonary circulation. J Saudi Heart Assoc. 2020;32(1):86–92. doi: 10.37616/2212-5043.1014 33154897 PMC7640615

[11] Gibbs JL, Rothman MT, Rees MR, Parsons JM, Blackburn ME, Ruiz CE. Stenting of the arterial duct: a new approach to palliation for pulmonary atresia. Br Heart J. 1992;67(3):240–5. doi: 10.1136/hrt.67.3.240 1372815 PMC1024799

[12] Gladman G, McCrindle BW, Williams WG, Freedom RM, Benson LN. The modified Blalock-Taussig shunt: clinical impact and morbidity in Fallot’s tetralogy in the current era. J Thorac Cardiovasc Surg. 1997;114(1):25–30. doi: 10.1016/S0022-5223(97)70113-2 9240290

[13] Santoro G, Capozzi G, Caianiello G, Palladino MT, Marrone C, Farina G, et al. Pulmonary artery growth after palliation of congenital heart disease with duct-dependent pulmonary circulation: arterial duct stenting versus surgical shunt. J Am Coll Cardiol. 2009;54(23):2180–6. doi: 10.1016/j.jacc.2009.07.043 19942090

[14] Amoozgar H, Cheriki S, Borzoee M, Ajami G, Soltani M, Ahmadipour M, et al. Short-term result of ductus arteriosus stent implantation compared with surgically created shunts. Pediatr Cardiol. 2012;33(8):1288–94. doi: 10.1007/s00246-012-0304-x 22447384

[15] Al Kindi FA, Al Kindi H, Maddali MM, Al Farqani A, Al Alawi K, Al Balushi A, et al. Comparing flow and pulmonary artery growth post-patent ductus arteriosus stenting in patients with ductal-dependent pulmonary flow using 4D magnetic resonance imaging. Eur Heart J Imaging Methods Pract. 2024;2(1):qyae044. doi: 10.1093/ehjimp/qyae044 39224104 PMC11367953

[16] Glatz AC, Petit CJ, Goldstein BH, Qureshi AM. Response by Glatz *et al*. to letter regarding article. Circulation. 2018;138:436–7. doi: 10.1161/CIRCULATIONAHA.118.035223 30571367

[17] McMullan DM, Permut LC, Jones TK, Johnston TA, Rubio AE. Modified Blalock-Taussig shunt versus ductal stenting for palliation of cardiac lesions with inadequate pulmonary blood flow. J Thorac Cardiovasc Surg. 2014;147(1):397–401. doi: 10.1016/j.jtcvs.2013.07.052 24071469

[18] Valencia E, Staffa SJ, Kuntz MT, Zaleski KL, Kaza AK, Maschietto N, et al. Transcatheter ductal stents versus surgical systemic-pulmonary artery shunts in neonates with congenital heart disease with ductal-dependent pulmonary blood flow: trends and associated outcomes from the pediatric health information system database. J Am Heart Assoc. 2023;12(17):e030528. doi: 10.1161/JAHA.123.030528 37589149 PMC10547312

