# Supplementary material for: Comparative outcomes of pulmonary artery changes in ductal-patency-dependent pulmonary circulation: Patent ductus arteriosus stent versus modified Blalock–Taussig shunt
Source: PLoS One. 2026 Sep 15;21(9):e0358266. doi: 10.1371/journal.pone.0358266 (PMC13577476; doi:10.1371/journal.pone.0358266)
Supplement: Supplementary file 1 [file pone.0358266.s002.docx]

Reviewer #2

1. Comment: As rightly mentioned in the Discussion, treatment allocation was not randomized. PDA stenting was preferentially performed in patients with biventricular physiology, whereas mBTS was preferentially performed in patients with univentricular physiology and unfavorable ductal anatomy. These baseline differences create a substantial risk of confounding by indication. Mortality and complication comparisons may therefore reflect underlying patient complexity rather than procedural superiority.

**Answer:** Thank you for this important comment. We agree that non-randomized treatment allocation introduces a substantial risk of confounding by indication and treatment-selection bias. As noted by the reviewer, PDA stenting was preferentially performed in patients with more favorable anatomy, particularly those with biventricular physiology and ductal morphology suitable for transcatheter intervention. Conversely, mBTS was more commonly performed in patients with univentricular physiology or unfavorable ductal anatomy, including markedly tortuous ducts, in whom PDA stenting was considered technically challenging or associated with a higher procedural risk.

We have revised the Discussion, particularly the Procedural Outcomes and Clinical Implications section, to clarify that differences in mortality and procedural complications should not be interpreted as evidence of procedural superiority. Rather, these observed differences may reflect baseline anatomical complexity, physiological instability, and surgical risk. We have also revised the Conclusion to present the findings more cautiously and to acknowledge that interpretation is limited by non-randomized treatment allocation and baseline differences in anatomical complexity.

Revised Manuscript Text, lines 344-365:

In this cohort, procedural complications were observed more frequently after mBTS than after PDA stenting, and the pattern of complications differed between treatment strategies. Complications after PDA stenting were primarily stent-related, whereas complications after mBTS were more commonly surgery-related, including pleural and thoracic complications. Although crude mortality was also higher in the mBTS group, this finding should be interpreted with caution because time-to-event analyses did not demonstrate a statistically significant survival difference. Kaplan–Meier analysis, Cox proportional hazards modeling, and 12-month restricted mean survival time analysis showed a higher observed survival probability after PDA stenting, but the between-group differences were not statistically significant.

These findings are consistent with prior comparative studies by McMullan et al. [17], Amoozgar et al. [14], and Valencia et al. [18], which generally reported broadly comparable early survival after PDA stenting and surgical shunting, without clear evidence of mortality superiority for either strategy. In our institutional practice, mBTS was more commonly selected for patients with univentricular physiology, markedly tortuous ductal morphology, or anatomy considered unsuitable or high risk for PDA stenting. Therefore, the observed differences in complications and crude mortality may partly reflect baseline anatomical complexity, physiological instability, procedural feasibility, and preprocedural risk rather than a direct treatment effect. Accordingly, the survival findings should be interpreted as observational associations and not as evidence of a causal survival advantage of PDA stenting over mBTS. Future prospective studies with standardized treatment selection, adequate adjustment for ductal morphology and ventricular physiology, and uniform follow-up are needed to better define the independent effect of palliation strategy on procedural safety and survival.

Revised Conclusion, lines 373–375

PDA stenting was associated with greater early pulmonary artery growth and fewer procedural complications in this cohort, although interpretation is limited by non-randomized treatment allocation and baseline differences in anatomical complexity.

2.Pulmonary artery growth was analyzed using independent t-tests at each follow-up point. Given the repeated measurements within individual patients, a repeated-measures approach (mixed-effects model or repeated-measures ANOVA) would be more appropriate.

**Answer:** Thank you for this important comment. We agree that pulmonary artery growth represents repeated measurements within individual patients and that independent t-tests at each follow-up point do not fully account for within-patient correlation over time. We have therefore revised the Statistical Analysis section and reanalyzed longitudinal pulmonary artery growth using linear mixed-effects models with a random intercept for each patient.

The revised manuscript now states in line 154-165

For longitudinal assessment of PA growth, the McGoon ratio and Nakata index were compared between the PDAs and mBTS groups at each prespecified time point (baseline, 2 months, and 12 months) using linear mixed-effects (random-intercept) models, with treatment group, follow-up time, and their interaction as fixed effects and a random intercept for each patient. This repeated-measures approach used all available observations under a missing-at-random assumption, thereby accommodating incomplete follow-up without restriction to complete cases. Between-group differences (PDAs minus mBTS) at each time point were estimated as model-based linear contrasts and are reported with 95% confidence intervals, and the overall group-by-time interaction was tested by likelihood-ratio test. To address potential confounding by indication, the longitudinal models were repeated with additional adjustment for ventricular physiology (biventricular vs univentricular) and its interaction with time.

3.No sample size or power calculation is reported.

**Answer:** Thank you for this comment. We have revised the Methods section to include the sample size calculation for the primary outcome of pulmonary artery growth, assessed by the Nakata index. The revised text has been added in lines 140–145.

**Revised manuscript**
Sample size was estimated for the primary outcome of pulmonary artery growth, assessed by the Nakata index. Using a two-independent-means formula with a two-sided test, prior reference data reported mean Nakata indices of 162 ± 26 and 194.7 ± 48 in infants undergoing ductal stenting and surgical shunting, respectively [10]. Assuming a two-sided alpha level of 0.05, 80% power, and a 2:1 allocation ratio, the required sample size was 41 patients. After accounting for approximately 10% incomplete data, the final estimated sample size was 46 patients

4.The data support the observation that PDA stenting was associated with: greater pulmonary artery growth at 2 months, lower observed complication rates and lower observed mortality. This conclusion may overstate the evidence because of baseline differences between treatment groups. Additionally, the study design doesn’t establish causality.

**Answer:** Thank you for this important comment. We agree that the retrospective observational design and baseline differences between treatment groups limit causal interpretation. Therefore, the observed differences in pulmonary artery growth, complications, and mortality should not be interpreted as evidence of procedural superiority or a causal treatment effect. We have revised the Discussion and Conclusion to present the findings more cautiously, emphasizing that interpretation is limited by non-randomized treatment allocation and baseline differences in anatomical complexity.

**Revised Conclusion, lines 373–375:**

PDA stenting was associated with greater early pulmonary artery growth and fewer procedural complications in this cohort, although interpretation is limited by non-randomized treatment allocation and baseline differences in anatomical complexity.

5 The data availability statement explains restrictions related to patient confidentiality. This is generally acceptable.

**Answer:** Thank you for this comment. We have retained the data availability statement as written, as it appropriately addresses restrictions related to patient confidentiality and ethical approval.

6.Address confounding by indication through additional statistical adjustment.

**Answer:** Thank you for this important comment. To address potential confounding by indication, we repeated the longitudinal pulmonary artery growth analyses with additional adjustment for ventricular physiology, classified as biventricular versus univentricular physiology, and its interaction with follow-up time. The between-group differences were essentially unchanged after adjustment. However, we acknowledge that residual confounding from other anatomical and clinical factors cannot be excluded, and the Discussion has been revised accordingly.

7.Reanalyze longitudinal pulmonary artery growth using methods appropriate for repeated measures.

**Answer:** Thank you for this comment. We agree that repeated measurements within individual patients should be analyzed using methods that account for within-patient correlation. We therefore replaced the per-timepoint independent t-tests with linear mixed-effects models using a random intercept for each patient. The Methods, Results, Abstract, and Discussion have been revised to reflect this repeated-measures analysis.

Revised Statistical Analysis section: **line 158-165**

This repeated-measures approach used all available observations under a missing-at-random assumption, thereby accommodating incomplete follow-up without restriction to complete cases. Between-group differences (PDAs minus mBTS) at each time point were estimated as model-based linear contrasts and are reported with 95% confidence intervals, and the overall group-by-time interaction was tested by likelihood-ratio test. To address potential confounding by indication, the longitudinal models were repeated with additional adjustment for ventricular physiology (biventricular vs univentricular) and its interaction with time.

Revised Results section: **line 215-250**

Pulmonary artery growth was evaluated using the McGoon ratio and Nakata index at baseline, 2 months, and 12 months after PDA stenting or mBTS (Figure 3 and Table 2). The mean McGoon ratio was 1.450 ± 0.285 at baseline. At baseline, the model-based between-group difference, calculated as PDA stenting minus mBTS, was −0.01 (95% CI, −0.13 to 0.11; p = 0.84). At 2 months, the mean McGoon ratio was 1.721 ± 0.325 in the PDA stenting group and 1.540 ± 0.194 in the mBTS group, with a model-based between-group difference of 0.18 (95% CI, 0.05 to 0.31; p = 0.005). At 12 months, the mean McGoon ratio was 2.080 ± 0.323 in the PDA stenting group and 1.930 ± 0.334 in the mBTS group, with a model-based between-group difference of 0.15 (95% CI, 0.02 to 0.29; p = 0.028). The longitudinal change in McGoon ratio differed between treatment groups (likelihood-ratio p = 0.018). After adjustment for ventricular physiology, the corresponding between-group differences were 0.00 at baseline (95% CI, −0.12 to 0.13; p = 0.97), 0.19 at 2 months (95% CI, 0.06 to 0.32; p = 0.005), and 0.16 at 12 months (95% CI, 0.02 to 0.30; p = 0.023).

The mean baseline Nakata index in the overall cohort was 134.322 ± 52.923 mm²/m² (Table 1). At baseline, the model-based between-group difference was 10.9 mm²/m² (95% CI, −15.7 to 37.4; p = 0.42). At 2 months, the mean Nakata index was 176.612 ± 58.080 mm²/m² in the PDA stenting group and 124.287 ± 41.292 mm²/m² in the mBTS group, with a model-based between-group difference of 51.2 mm²/m² (95% CI, 23.0 to 79.5; p < 0.001). At 12 months, the mean Nakata index was 237.537 ± 79.135 mm²/m² in the PDA stenting group and 236.569 ± 84.913 mm²/m² in the mBTS group, with a model-based between-group difference of −2.0 mm²/m² (95% CI, −31.7 to 27.8; p = 0.90). The longitudinal change in Nakata index differed between treatment groups (likelihood-ratio p = 0.009). After adjustment for ventricular physiology, the corresponding between-group differences were 8.0 mm²/m² at baseline (95% CI, −19.5 to 35.6; p = 0.57), 51.9 mm²/m² at 2 months (95% CI, 22.8 to 81.0; p < 0.001), and −3.5 mm²/m² at 12 months (95% CI, −34.4 to 27.5; p = 0.83). The LPA/RPA ratio did not differ at baseline or at 2 months but was significantly lower in the PDAs group at 12 months (Table 3), indicating progressive branch asymmetry despite similar global PA dimensions.

8.Clarify antiplatelet regimens in the Methods section and reconcile with statements in the Discussion.

**Answer:** Thank you for this comment. We have revised the Methods section to clarify the post-procedural antithrombotic regimens used in each treatment group. Specifically, lines 123–129 now state that patients undergoing PDA stenting received intravenous heparin for at least 24 hours, followed by dual antiplatelet therapy with aspirin and clopidogrel for 6 months, and then aspirin monotherapy until definitive surgical repair or the next-stage palliation. Patients undergoing mBTS received intravenous heparin for at least 24 hours postoperatively, followed by aspirin monotherapy at 3–5 mg/kg/day until definitive surgical repair or the next-stage palliation. The Discussion section has also been revised to ensure consistency with the Methods section in line 316-317.

9.Include confidence intervals for key pulmonary artery growth outcomes.

**Answer:** Thank you for this comment. We have revised Figure 3 to include 95% confidence intervals for the key pulmonary artery growth outcomes. The figure now presents model-based mean estimates with 95% confidence intervals derived from linear mixed-effects models for both the McGoon ratio and Nakata index at baseline, 2 months, and 12 months. The Results section and Table 3 have also been updated to report model-based between-group differences with 95% confidence intervals.

10.Interpret conclusions about mortality benefit with caution.

**Answer:** Thank you for this comment. We have revised the conclusion to avoid implying a direct mortality benefit of PDA stenting. Specifically, in lines 373–375, we now state that PDA stenting was associated with greater early pulmonary artery growth and fewer procedural complications in this cohort, although interpretation is limited by non-randomized treatment allocation and baseline differences in anatomical complexity.

11.Ethics approval from both participating institutions is clearly reported. Waiver of informed consent is appropriately justified for retrospective anonymized data analysis.

Answer: The study was approved by the institutional ethics committees of both participating centers: Songklanagarind Hospital (REC.66-499-1-1) and Queen Sirikit National Institute of Child Health (REC.070/2567). The requirement for informed consent was waived because the study involved retrospective analysis of anonymized data. The study was conducted in accordance with the principles of the Declaration of Helsinki.
