# Supplementary material for: Comparative outcomes of pulmonary artery changes in ductal-patency-dependent pulmonary circulation: Patent ductus arteriosus stent versus modified Blalock–Taussig shunt
Source: PLoS One. 2026 Sep 15;21(9):e0358266. doi: 10.1371/journal.pone.0358266 (PMC13577476; doi:10.1371/journal.pone.0358266)
Supplement: Supplementary file 2 [file pone.0358266.s003.docx]

Response to the Academic Editor and Reviewers

Manuscript ID: PONE-D-26-13265

Manuscript Title: Comparative outcomes of pulmonary artery changes in ductal-patency dependent pulmonary circulation: patent ductus arteriosus stent versus modified Blalock–Taussig shunt

To: Atnafu Mekonnen Tekleab, M.D., MPH

Academic Editor, PLOS ONE

Thank you for your helpful comments and guidance. We have revised the manuscript accordingly.

***Comment 1***: Please write the abstract section in a structured form (Background/Context, Methods, Results, and Conclusions).

**Response:** Thank you for this suggestion. We have revised the Abstract into a structured format with the following headings: Background/Context, Methods, Results, and Conclusions.

**Abstract**

**Background:** Neonates with ductal-dependent pulmonary blood flow (DP-PBF) require timely palliation to maintain pulmonary perfusion. Patent ductus arteriosus (PDA) stenting has emerged as a less invasive alternative to modified Blalock–Taussig shunt (mBTS), but comparative data on longitudinal pulmonary artery growth and clinical outcomes remain limited.

**Methods:** This multicenter retrospective cohort study included infants with DP-PBF who underwent PDA stenting or mBTS as first-stage palliation at Queen Sirikit National Institute of Child Health and Songklanagarind Hospital, Thailand, between January 2017 and December 2022. Pulmonary artery growth was assessed using the McGoon ratio and Nakata index at baseline, 2 months, and 12 months. Longitudinal changes were analyzed using linear mixed-effects models with adjustment for ventricular physiology. Procedural complications were compared, and mortality was evaluated using Kaplan–Meier analysis, Cox regression, and 12-month restricted mean survival time.

**Results:** Eighty-six infants were included: 41 underwent PDA stenting and 45 underwent mBTS. Baseline pulmonary artery measurements were comparable, although diagnostic composition differed between groups. At 2 months, PDA stenting was associated with greater pulmonary artery growth than mBTS, with a higher McGoon ratio (mean difference, 0.18; 95% CI, 0.05–0.31; p = 0.005) and Nakata index (mean difference, 51.2 mm²/m²; 95% CI, 23.0–79.5; p < 0.001). By 12 months, the Nakata index was similar between groups (p = 0.90), whereas a statistically significant between-group difference in McGoon ratio remained (mean difference, 0.15; 95% CI, 0.02–0.29; p = 0.028). Procedural complications were less frequent after PDA stenting than after mBTS (17.1% vs 57.8%; risk ratio for mBTS vs PDA stenting, 3.38; 95% CI, 1.65–6.95; p = 0.001). Although mortality was numerically lower after PDA stenting, time-to-event analyses did not show a statistically significant survival difference.

**Conclusions:** In this cohort, PDA stenting was associated with greater early pulmonary artery growth and fewer observed procedural complications compared with mBTS. By 12 months, the Nakata index was similar between groups, whereas a statistically significant difference in McGoon ratio remained. These findings should be interpreted cautiously because of non-randomized treatment allocation, differences in diagnostic composition, and potential residual confounding.

***Comment 2***: Lines 38–39 read as “…whereas a small McGoon ratio difference persisted (p = 0.028).” This statement is vague and does not sound scientific. You have already set p-value < 0.05 to be statistically significant. Then, what is the implication of saying “…a small McGoon ratio difference persisted…” while the p-value is < 0.05?

**Response:** Thank you for this important clarification. We agree that the original wording was vague. We have revised this sentence to report the finding more precisely using the estimated between-group difference and 95% confidence interval. The revised sentence now reads: “By 12 months, the Nakata index was similar between groups (p = 0.90), whereas a statistically significant between-group difference in McGoon ratio remained (mean difference, 0.15; 95% CI, 0.02–0.29; p = 0.028). Procedural complications were less frequent after PDA stenting than after mBTS (17.1% vs 57.8%; risk ratio for mBTS vs PDA stenting, 3.38; 95% CI, 1.65–6.95; p = 0.001). Although mortality was numerically lower after PDA stenting, time-to-event analyses did not show a statistically significant survival difference.”

We have also revised the Discussion section in Lines 325–332 to ensure consistency with the revised Abstract and Results. By 12 months, the Nakata index was similar between groups, whereas a statistically significant between-group difference in McGoon ratio remained. These findings suggest that PDA stenting was associated with greater early pulmonary artery growth, followed by attenuation of the between-group difference over time. The similar Nakata index at 12 months indicates largely comparable indexed pulmonary artery size, while the remaining McGoon ratio difference may reflect residual differences in branch pulmonary artery dimensions relative to the descending aorta. Therefore, the 12 month findings should be interpreted as partial convergence of pulmonary artery growth rather than complete equivalence between treatment groups

***Comment 3***: I am happy that you have revised the statistical test methods employed. Please state if your data set fulfills the criteria to apply parametric tests such as Cox regression and Kaplan–Meier survival analysis models.

**Response**: Thank you for this comment. We have revised the Statistical Analysis section to clarify the suitability of the dataset for survival analysis in Lines 175–182: “Mortality was evaluated descriptively and using time-to-event analyses. Overall survival was defined as the time from the index procedure to death from any cause or censoring at the last documented follow-up. Because the index procedure date, date of death, and censoring or last follow-up date were available, Kaplan–Meier curves were generated and compared using the log-rank test. Cox proportional hazards models were used to estimate hazard ratios for mortality, with additional adjustment for ventricular physiology. The proportional hazards assumption was assessed before Cox regression. Restricted mean survival time was calculated up to 12 months to summarize survival over the prespecified follow-up interval.”

Regarding the reference list, we have reviewed all cited references for completeness, accuracy, and publication status. No retracted articles were identified among the cited references; therefore, no changes related to retracted publications were required.

Kind regards,

Kanjarut Wongwaitaweewong, MD
