# Supplementary material for: Comparative outcomes of pulmonary artery changes in ductal-patency-dependent pulmonary circulation: Patent ductus arteriosus stent versus modified Blalock–Taussig shunt
Source: PLoS One. 2026 Sep 15;21(9):e0358266. doi: 10.1371/journal.pone.0358266 (PMC13577476; doi:10.1371/journal.pone.0358266)
Supplement: Peer review history

# Peer review: Decision Letter 0

**DOI:** 10.1371/journal.pone.0358266.r001

**Authors:** Atnafu Mekonnen Tekleab

10 Jun 2026

Dear Dr. Wongwaitaweewong,

Thank you for submitting your manuscript to PLOS ONE. After careful consideration, we feel that it has merit but does not fully meet PLOS ONE’s publication criteria as it currently stands. Therefore, we invite you to submit a revised version of the manuscript that addresses the points raised during the review process.

==============================

**ACADEMIC EDITOR:**

I would like to thank the authors for their effort in trying to address a clinically relevant question regarding the comparative effectiveness of patent ductus arteriosus stenting (PDAs) vs modified Blalock-Taussig shunt (mBTS). In addition to addressing the comments raised by the reviewers, I would like to request the authors to address the following issues.

Major concern:

- The two groups were not comparable at baseline since they had different physiology which affects survival and response to the treatment they received. To me, this is just like “comparing apple with orange”. I appreciate that the authors have acknowledged this issue, but the analyses do not adequately adjust for these important differences.

Additional comments:

Introduction section:

- Lines 74-77: “The primary objective of this study was to assess the effectiveness of PDAs in facilitating PA growth….”. This statement is not in line with the stated objective of the study in other sections of the manuscript “…comparative effectiveness of PDAs vs mBTS..”.Please do revision accordingly.

Methods section:

- Lines 104-134: Procedure selection and techniques: This section is not important for the readers of this article. You may briefly mention the procedures and then cite reference/s so that readers can refer it if they want to know the details since such procedures follow standard protocols across different settings.
- Please remove Fig 1 since it doesn’t add value to the research question that you have tried to address.
- Lines 147-147: The rationale for comparing outcomes only at the pre-specified time points of baseline, 2 months, and 12 months is unclear. Given that this was a cohort study, it would be helpful to explain why outcomes were not analyzed over the entire follow-up period (e.g., within 1 year) using time-to-event methods. Assessing outcomes at fixed time points may not fully capture the timing and occurrence of clinically important events.

Result section:

- Lines 161-166: There is significant attrition rate in both groups (15/45 vs 11/41). However, the reason for attrition in the two groups is different (attrition due to death: 12/15 for the mBTS group vs attrition due to death 2/11 for the PDAs group). This might reflect the difference in the base line characteristics of the two groups implying comparing the treatment outcome in the two groups has methodological flaws. Also, this is a potential cause for attrition bias and it is not controlled in the analysis and not discussed in the discussion section. Please explain.
- Line 207: Does the following statement refers to mBTS or PDAs or a combination of the two population group? “The mean Nakata index was 134.322 ± 52.923 mm²/m² at baseline.” Please clarify.
- Mortality is a key outcome measure in the study, yet only crude proportions are presented. (death over-time not depicted)
- What about using Kaplan-Meier survival curves and Cox proportional hazards/log rank analysis?

Discussion section:

- Lines 287-289: Authors claimed that the “…the higher mortality in MBTS group may be due to differences in underlying patient complexity rather than a direct causal effect of the surgical approach itself.”. I am happy that the authors have mentioned such significant limitation of the study. However, I feel that the statistical test findings which were performed without adequate controlling/adjustment may still confuse readers. I would like to ask the authors to make necessary revisions in the statistical tests they applied and the discussion as well conclusion sections (over-stated).
- What is the clinical relevance of significant pulmonary artery growth difference between the two groups at 2 months? Please give explanation.

==============================

Please submit your revised manuscript by Jul 25 2026 11:59PM. If you will need more time than this to complete your revisions, please reply to this message or contact the journal office at plosone@plos.org. When you're ready to submit your revision, log on to https://www.editorialmanager.com/pone/ and select the 'Submissions Needing Revision' folder to locate your manuscript file.

- A letter that responds to each point raised by the academic editor and reviewer(s). You should upload this letter as a separate file labeled 'Response to Reviewers'.
- A marked-up copy of your manuscript that highlights changes made to the original version. You should upload this as a separate file labeled 'Revised Manuscript with Track Changes'.
- An unmarked version of your revised paper without tracked changes. You should upload this as a separate file labeled 'Manuscript'.

If you would like to make changes to your financial disclosure, please include your updated statement in your cover letter. Guidelines for resubmitting your figure files are available below the reviewer comments at the end of this letter.

If applicable, we recommend that you deposit your laboratory protocols in protocols.io to enhance the reproducibility of your results. Protocols.io assigns your protocol its own identifier (DOI) so that it can be cited independently in the future. For instructions see: https://journals.plos.org/plosone/s/submission-guidelines#loc-laboratory-protocols. Additionally, PLOS ONE offers an option for publishing peer-reviewed Lab Protocol articles, which describe protocols hosted on protocols.io. Read more information on sharing protocols at https://plos.org/protocols?utm_medium=editorial-email&utm_source=authorletters&utm_campaign=protocols.

As the corresponding author, your ORCID iD is verified in the submission system and will appear in the published article. PLOS supports the use of ORCID, and we encourage all coauthors to register for an ORCID iD and use it as well. Please encourage your coauthors to verify their ORCID iD within the submission system before final acceptance, as unverified ORCID iDs will not appear in the published article. *Only*  the individual author can complete the verification step; PLOS staff *cannot*  verify ORCID iDs on behalf of authors.

We look forward to receiving your revised manuscript.

Kind regards,

Atnafu Mekonnen Tekleab, M.D, MPH

Academic Editor

PLOS One

Journal Requirements:

When submitting your revision, we need you to address these additional requirements.

1. Please ensure that your manuscript meets PLOS ONE's style requirements, including those for file naming. The PLOS ONE style templates can be found at

https://journals.plos.org/plosone/s/file?id=wjVg/PLOSOne_formatting_sample_main_body.pdf and https://journals.plos.org/plosone/s/file?id=ba62/PLOSOne_formatting_sample_title_authors_affiliations.pdf

2. In the online submission form, you indicated that the data underlying the results presented in this study cannot be shared publicly due to patient confidentiality and institutional data protection regulations. Data are available from the Human Research Ethics Committee of Prince of Songkla University for researchers who meet the criteria for access to confidential data upon reasonable request.

All PLOS journals now require all data underlying the findings described in their manuscript to be freely available to other researchers, either 1. In a public repository, 2. Within the manuscript itself, or 3. Uploaded as supplementary information.

This policy applies to all data except where public deposition would breach compliance with the protocol approved by your research ethics board. If your data cannot be made publicly available for ethical or legal reasons (e.g., public availability would compromise patient privacy), please explain your reasons on resubmission and your exemption request will be escalated for approval.

3. We note that Figure 2 in your submission contain copyrighted images. All PLOS content is published under the Creative Commons Attribution License (CC BY 4.0), which means that the manuscript, images, and Supporting Information files will be freely available online, and any third party is permitted to access, download, copy, distribute, and use these materials in any way, even commercially, with proper attribution. For more information, see our copyright guidelines: http://journals.plos.org/plosone/s/licenses-and-copyright.

We require you to either (1) present written permission from the copyright holder to publish these figures specifically under the CC BY 4.0 license, or (2) remove the figures from your submission:

a. You may seek permission from the original copyright holder of Figure 2 to publish the content specifically under the CC BY 4.0 license.

We recommend that you contact the original copyright holder with the Content Permission Form (http://journals.plos.org/plosone/s/file?id=7c09/content-permission-form.pdf) and the following text:

“I request permission for the open-access journal PLOS ONE to publish XXX under the Creative Commons Attribution License (CCAL) CC BY 4.0 (http://creativecommons.org/licenses/by/4.0/). Please be aware that this license allows unrestricted use and distribution, even commercially, by third parties. Please reply and provide explicit written permission to publish XXX under a CC BY license and complete the attached form.”

Please upload the completed Content Permission Form or other proof of granted permissions as an "Other" file with your submission.

In the figure caption of the copyrighted figure, please include the following text: “Reprinted from [ref] under a CC BY license, with permission from [name of publisher], original copyright [original copyright year].”

b. If you are unable to obtain permission from the original copyright holder to publish these figures under the CC BY 4.0 license or if the copyright holder’s requirements are incompatible with the CC BY 4.0 license, please either i) remove the figure or ii) supply a replacement figure that complies with the CC BY 4.0 license. Please check copyright information on all replacement figures and update the figure caption with source information. If applicable, please specify in the figure caption text when a figure is similar but not identical to the original image and is therefore for illustrative purposes only.

4. If the reviewer comments include a recommendation to cite specific previously published works, please review and evaluate these publications to determine whether they are relevant and should be cited. There is no requirement to cite these works unless the editor has indicated otherwise.

[Note: HTML markup is below. Please do not edit.]

Reviewers' comments:

Reviewer's Responses to Questions

**Comments to the Author**

1. Is the manuscript technically sound, and do the data support the conclusions?

Reviewer #1: Partly

Reviewer #2: Partly

**********

2. Has the statistical analysis been performed appropriately and rigorously?

Reviewer #1: Yes

Reviewer #2: Yes

**********

3. Have the authors made all data underlying the findings in their manuscript fully available?

The PLOS Data policy

Reviewer #1: Yes

Reviewer #2: Yes

**********

4. Is the manuscript presented in an intelligible fashion and written in standard English?

Reviewer #1: Yes

Reviewer #2: Yes

**********

Reviewer #1: Selection Bias / Confounding

This is the major limitation of the study.

You have clearly stated:

• PDAs preferentially used in biventricular physiology

• mBTS more common in uni-ventricular physiology and tortuous ducts

This creates substantial baseline treatment-selection bias.

Mortality and complication differences may reflect: underlying anatomy or surgical complexity, than treatment superiority.

Mortality Interpretation

Your conclusion may overstate PDAs superiority.

Current wording: “PDAs results in greater early PA growth and lower complication burden...”

But avoid implying definitive superiority.

Suggested revision

“PDAs was associated with greater early PA growth and fewer procedural complications in this cohort, although interpretation is limited by non-randomized treatment allocation and differences in baseline anatomical complexity.”

Reviewer #2: Reviewer Comments

Manuscript Title: Comparative outcomes of pulmonary artery changes in ductal-patency dependent pulmonary circulation: patent ductus arteriosus stent versus modified Blalock-Taussig shunt

Overall Assessment: The topic is clinically important, particularly given the increasing adoption of transcatheter palliation strategies. The manuscript is generally well structured and clearly written. However, important methodological limitations, particularly related to treatment-selection bias and statistical analysis, limit the strength of the conclusions. The manuscript would benefit from revision before publication.

Major concerns include:

� As rightly mentioned in the discussion part treatment allocation was not random. PDA stenting was preferentially performed in patients with biventricular physiology, while mBTS was preferentially performed in patients with univentricular physiology and unfavorable ductal anatomy. These baseline differences create substantial risk of confounding by indication. Mortality and complication comparisons may therefore reflect underlying patient complexity rather than procedural superiority.

� Pulmonary artery growth was analyzed using independent t-tests at each follow-up point. Given the repeated measurements within individual patients, a repeated-measures approach (mixed-effects model or repeated-measures ANOVA) would be more appropriate.

� No sample size or power calculation is reported.

� The data support the observation that PDA stenting was associated with: greater pulmonary artery growth at 2 months, lower observed complication rates and lower observed mortality. This conclusion may overstate the evidence because of baseline differences between treatment groups. Additionally, the study design doesn’t establish causality.

� The data availability statement explains restrictions related to patient confidentiality. This is generally acceptable.

� Address confounding by indication through additional statistical adjustment.

� Reanalyze longitudinal pulmonary artery growth using methods appropriate for repeated measures.

� Clarify antiplatelet regimens in the Methods section and reconcile with statements in the Discussion.

� Include confidence intervals for key pulmonary artery growth outcomes.

� Interpret conclusions about mortality benefit with caution.

� Ethics approval from both participating institutions is clearly reported. Waiver of informed consent is appropriately justified for retrospective anonymized data analysis.

**********

what does this mean?). If published, this will include your full peer review and any attached files.

If you choose “no”, your identity will remain anonymous but your review may still be made public.

**Do you want your identity to be public for this peer review?**  For information about this choice, including consent withdrawal, please see our Privacy Policy

Reviewer #1: **Yes:** Dr. Temesgen Tsega, Consultant pediatric cardiologist

Reviewer #2: No

**********

[NOTE: If reviewer comments were submitted as an attachment file, they will be attached to this email and accessible via the submission site. Please log into your account, locate the manuscript record, and check for the action link "View Attachments". If this link does not appear, there are no attachment files.]

To ensure your figures meet our technical requirements, please review our figure guidelines: https://journals.plos.org/plosone/s/figures

You may also use PLOS’s free figure tool, NAAS, to help you prepare publication quality figures: https://journals.plos.org/plosone/s/figures#loc-tools-for-figure-preparation.

NAAS will assess whether your figures meet our technical requirements by comparing each figure against our figure specifications.

# Author comment: Author response to Decision Letter 1

**DOI:** 10.1371/journal.pone.0358266.r002

11 Aug 2026

The manuscript has been rechecked, and the necessary changes have been made in accordance with the reviewers’ suggestions. The revisions in the manuscript are highlighted in yellow. The responses to all comments have been prepared and attached herewith.

Thank you for your consideration. I look forward to hearing from you.

Attachment Submitted filename: Response to Reviewer2.docx

# Editor report: Decision Letter 1

**DOI:** 10.1371/journal.pone.0358266.r003

**Authors:** Atnafu Mekonnen Tekleab

24 Aug 2026

PLOS One

Dear Dr.  Wongwaitaweewong,

Thank you for submitting your manuscript to PLOS One. After careful consideration, we feel that it has merit but does not fully meet PLOS One’s publication criteria as it currently stands. Therefore, we invite you to submit a revised version of the manuscript that addresses the points raised during the review process.

==============================

**ACADEMIC EDITOR:**

Thank you for your detailed response to address the comments raised by the reviewers and me. Please would you address the following few comments:

- Please write the abstract section in a structured form (Background/context, Methods, result and conclusion)
- lines 38-39 read as “…whereas a small McGoon ratio difference persisted (p=0.028)”. This statement is vague and doesn’t sound scientific. You have already set P-value <0.05 to be statistically significant. Then, what is the implication of saying “… a small McGoon ratio difference persisted…” while the p-value is <0.05?
- I am happy that you have revised the statistical test methods employed. Please state if your data set fulfills the criteria to apply parametric tests such as Cox regression and Kaplan Meier survival analysis models.

============================

Please submit your revised manuscript by Oct 08 2026 11:59PM. If you will need more time than this to complete your revisions, please reply to this message or contact the journal office at plosone@plos.org. When you're ready to submit your revision, log on to https://www.editorialmanager.com/pone/ and select the 'Submissions Needing Revision' folder to locate your manuscript file.

Please include the following items when submitting your revised manuscript:

- A letter that responds to each point raised by the academic editor and reviewer(s). You should upload this letter as a separate file labeled 'Response to Reviewers'.
- A marked-up copy of your manuscript that highlights changes made to the original version. You should upload this as a separate file labeled 'Revised Manuscript with Track Changes'.
- An unmarked version of your revised paper without tracked changes. You should upload this as a separate file labeled 'Manuscript'.

If you would like to make changes to your financial disclosure, please include your updated statement in your cover letter. Guidelines for resubmitting your figure files are available below the reviewer comments at the end of this letter.

If applicable, we recommend that you deposit your laboratory protocols in protocols.io to enhance the reproducibility of your results. Protocols.io assigns your protocol its own identifier (DOI) so that it can be cited independently in the future. For instructions see: https://journals.plos.org/plosone/s/submission-guidelines#loc-laboratory-protocols. Additionally, PLOS One offers an option for publishing peer-reviewed Lab Protocol articles, which describe protocols hosted on protocols.io. Read more information on sharing protocols at https://plos.org/protocols?utm_medium=editorial-email&utm_source=authorletters&utm_campaign=protocols.

As the corresponding author, your ORCID iD is verified in the submission system and will appear in the published article. PLOS supports the use of ORCID, and we encourage all coauthors to register for an ORCID iD and use it as well. Please encourage your coauthors to verify their ORCID iD within the submission system before final acceptance, as unverified ORCID iDs will not appear in the published article. *Only*  the individual author can complete the verification step; PLOS staff *cannot*  verify ORCID iDs on behalf of authors.

We look forward to receiving your revised manuscript.

Kind regards,

Atnafu Mekonnen Tekleab, M.D, MPH

Academic Editor

PLOS One

Journal Requirements:

1. If the reviewer comments include a recommendation to cite specific previously published works, please review and evaluate these publications to determine whether they are relevant and should be cited. There is no requirement to cite these works unless the editor has indicated otherwise.

2. Please review your reference list to ensure that it is complete and correct. If you have cited papers that have been retracted, please include the rationale for doing so in the manuscript text, or remove these references and replace them with relevant current references. Any changes to the reference list should be mentioned in the rebuttal letter that accompanies your revised manuscript. If you need to cite a retracted article, indicate the article’s retracted status in the References list and also include a citation and full reference for the retraction notice.

[Note: HTML markup is below. Please do not edit.]

[NOTE: If reviewer comments were submitted as an attachment file, they will be attached to this email and accessible via the submission site. Please log into your account, locate the manuscript record, and check for the action link "View Attachments". If this link does not appear, there are no attachment files.]

To ensure your figures meet our technical requirements, please review our figure guidelines: https://journals.plos.org/plosone/s/figures

You may also use PLOS’s free figure tool, NAAS, to help you prepare publication quality figures: https://journals.plos.org/plosone/s/figures#loc-tools-for-figure-preparation.

NAAS will assess whether your figures meet our technical requirements by comparing each figure against our figure specifications.

# Author comment: Author response to Decision Letter 2

**DOI:** 10.1371/journal.pone.0358266.r004

27 Aug 2026

Thank you for your helpful comments and guidance. We have revised the manuscript accordingly.

Attachment Submitted filename: Response to Reviewers.docx

# Editor report: Decision Letter 2

**DOI:** 10.1371/journal.pone.0358266.r005

**Authors:** Atnafu Mekonnen Tekleab

30 Aug 2026

Comparative outcomes of pulmonary artery changes in ductal-patency dependent pulmonary circulation: patent ductus arteriosus stent versus modified Blalock-Taussig shunt

PONE-D-26-13265R2

Dear Dr. Wongwaitaweewong,

We’re pleased to inform you that your manuscript has been judged scientifically suitable for publication and will be formally accepted for publication once it meets all outstanding technical requirements.

Within one week, you’ll receive an e-mail detailing the required amendments. When these have been addressed, you’ll receive a formal acceptance letter and your manuscript will be scheduled for publication.

An invoice will be generated when your article is formally accepted. Please note, if your institution has a publishing partnership with PLOS and your article meets the relevant criteria, all or part of your publication costs will be covered. Please make sure your user information is up-to-date by logging into Editorial Manager at Editorial Manager® and clicking the ‘Update My Information' link at the top of the page. For questions related to billing, please contact billing support.

If your institution or institutions have a press office, please notify them about your upcoming paper to help maximize its impact. If they’ll be preparing press materials, please inform our press team as soon as possible -- no later than 48 hours after receiving the formal acceptance. Your manuscript will remain under strict press embargo until 2 pm Eastern Time on the date of publication. For more information, please contact onepress@plos.org.

Kind regards,

Atnafu Mekonnen Tekleab, M.D, MPH

Academic Editor

PLOS One

Additional Editor Comments (optional):

Reviewers' comments:

# Editor report: Acceptance letter

**DOI:** 10.1371/journal.pone.0358266.r006

**Authors:** Atnafu Mekonnen Tekleab

PONE-D-26-13265R2

PLOS One

Dear Dr. Wongwaitaweewong,

I'm pleased to inform you that your manuscript has been deemed suitable for publication in PLOS One. Congratulations! Your manuscript is now being handed over to our production team.

At this stage, our production department will prepare your paper for publication. This includes ensuring the following:

* All references, tables, and figures are properly cited

* All relevant supporting information is included in the manuscript submission,

* There are no issues that prevent the paper from being properly typeset

You will receive further instructions from the production team, including instructions on how to review your proof when it is ready. Please keep in mind that we are working through a large volume of accepted articles, so please give us a few days to review your paper and let you know the next and final steps.

Lastly, if your institution or institutions have a press office, please let them know about your upcoming paper now to help maximize its impact. If they'll be preparing press materials, please inform our press team within the next 48 hours. Your manuscript will remain under strict press embargo until 2 pm Eastern Time on the date of publication. For more information, please contact onepress@plos.org.

You will receive an invoice from PLOS for your publication fee after your manuscript has reached the completed accept phase. If you receive an email requesting payment before acceptance or for any other service, this may be a phishing scheme. Learn how to identify phishing emails and protect your accounts at https://explore.plos.org/phishing.

If we can help with anything else, please email us at customercare@plos.org.

Thank you for submitting your work to PLOS One and supporting open access.

Kind regards,

PLOS One Editorial Office Staff

on behalf of

Dr. Atnafu Mekonnen Tekleab

Academic Editor

PLOS One
